# The co-application of arbuscular mycorrhizal fungus and *Trichoderma* on anthracnose disease in common vetch

**DOI:** 10.3389/frmbi.2025.1654549

**Published:** 2025-09-15

**Authors:** Jia He, Faxi Li, Rongchun Zheng, Meiting Bai, Ping Wang, Tingyu Duan

**Affiliations:** ^1^ College of Forestry and Prataculture, Ningxia University, Yinchuan, China; ^2^ College of Pastoral Agriculture Science and Technology, State Key Laboratory of Herbage Improvement and Grassland Agro-ecosystems, Engineering Research Center of Grassland Industry, Ministry of Education, Gansu Tech Innovation Center of Western China Grassland Industry, Key Laboratory of Grassland Livestock Industry Innovation, Ministry of Agriculture and Rural Affairs, Lanzhou University, Lanzhou, China; ^3^ Gansu Open University, Lanzhou, China; ^4^ Dingxi Vocational and Technical College, Dingxi, China

**Keywords:** *Vicia sativa*, anthracnose, biological control, defense response, physiological responses

## Abstract

**Introduction:**

Common vetch (*Vicia sativa*) is an important legume used for forage and green manure. Anthracnose caused by *Colletotrichum spinaciae* is a significant disease affecting common vetch, resulting in significant damage and yield reductions. Furthermore, there is a lack of effective control methods for this disease.

**Methods:**

This study evaluated the control of anthracnose in *V. sativa* under greenhouse conditions, focusing on the efficacy of 25% pyraclostrobin, the arbuscular mycorrhizal (AM) fungus *Glomus tortuosum*, and *Trichoderma longibrachiatum*, both individually and in combination.

**Results:**

The results showed that 25% pyraclostrobin, *G. tortuosum*, and *T. longibrachiatum* both individually and in combination reduced the incidence of anthracnose by 53.85%, 34.62%, 34.62%, and 15.39%, respectively. Correspondingly, the disease index decreased by 68.97%, 34.48%, 32.76%, and 20.69%. Notably, the application of *G. tortuosum* and *T. longibrachiatum* alone enhanced common vetch defense enzyme activities of peroxidase, catalase, superoxide dismutase, and polyphenol oxidase by 23.57% and 22.10%, 27.12% and 26.76%, 21.54% and 19.33%, and 35.79% and 34.35%, respectively (*P* < 0.05). Moreover, the application of AM fungi and *Trichoderma* led to increased activities of soil urease, catalase, and neutral phosphatase by 12.77% to 111.17%, as well as improved nitrogen and phosphorus uptake by 12.12% to 13.88% and 13.91% to 35.79%, respectively.

**Discussiom:**

Our findings highlight that *G. tortuosum* and *T. longibrachiatum* can effectively induce resistance against anthracnose in common vetch, demonstrating significant control efficacy.

## 1 Introduction

Common vetch serves not only as an exceptional forage crop but also as a widely used green manure in agricultural production. The growth of *Vicia sativa* is threatened by numerous biotic and abiotic factors, with diseases emerging as a critical constraint on both quality and yield (Xu and Li, 2015). In recent years, anthracnose caused by *Colletotrichum* spp., particularly *Colletotrichum spinaciae*, has become prevalent in common vetch production, primarily affecting the leaves, stems, and pods. In severe cases, the disease can lead to leaf dieback and even plant mortality (Wang et al., 2019), resulting in significant declines in yield, quality, and economic benefits. This decline poses challenges to the development of agriculture and animal husbandry in regions where *V. sativa* is cultivated.

Despite the severity of diseases affecting common vetch, research on effective control strategies remains limited, primarily concentrating on the use of disease-resistant varieties and agricultural management practices (Fernández-aparicio et al., 2009; Rico et al., 2006). While research on disease control in common vetch has gradually increased in recent years, there is still a lack of targeted, systematic, and comprehensive control measures. Therefore, it is imperative to explore effective methods for the prevention and control of anthracnose in this crop and to identify common vetch varieties with inherent disease resistance.

Pyraclostrobin, classified as a methoxyacrylate fungicide, is recognized for its high efficiency, low toxicity, broad-spectrum activity, and systemic properties. It has demonstrated effective control against *Colletotrichum destructivum* in alfalfa (*Medicago sativa*) and can effectively inhibit the mycelial growth of *C. spinaciae*, offering substantial prevention and treatment against *C. destructivum* in alfalfa (Vasi et al., 2019). Additionally, the combined application of pyraclostrobin with other agents, such as a mixture of 32.5% benomyl and 25% pyraclostrobin, as well as combinations with 50% carbendazim, has shown a field preventive effect exceeding 63% against anthracnose in common vetch (Li F. X. et al., 2021). However, despite these advantages, chemical agents can exert pressure on the environment, particularly in grassland ecosystems where aboveground tissues are directly consumed by livestock. As a result, disease prevention and control often face significant limitations. Due to the secondary and supportive role of chemical control measures, these agents are primarily utilized in primary seed fields or experimental settings. Consequently, it is urgent to explore alternative control methods for managing diseases in common vetch. Furthermore, the emergence of pyraclostrobin resistance can be a problem. Field resistance to pyraclostrobin has been reported in populations of *Alternaria solani* causing early blight in potatoes, linked to specific point mutations (e.g., F129L) in the cytochrome b gene (Pasche and Gudmestad, 2008). The cytochrome b gene-based assay is used for monitoring the resistance of *Colletotrichum* spp. to pyraclostrobin (Isa and Kim, 2022).

Biological control is an important complement to chemical pesticides, providing benefits such as low cost, environmental friendliness, and sustainability. Biocontrol microorganisms, notably arbuscular mycorrhizal (AM) fungi and *Trichoderma*, are extensively employed in the prevention and control of plant diseases (Castellanos-Morales et al., 2012; Palani and Lalithakumari, 1999). AM fungi enhance plant growth performance, compete with pathogens for nutrients and infection sites, enrich the root microbiome, and modify root system structure to bolster plant resistance against pathogens (El-Sharkawy et al., 2018). Research has demonstrated that inoculation with AM fungi significantly suppresses the occurrence of spring black stem disease (*Phoma medicaginis*) in *Medicago sativa* (Li Y. D. et al., 2021).

*Trichoderma* employs several biocontrol mechanisms, including hyperparasitism toward pathogenic fungi, competition with pathogens for survival resources and space, synthesis of antimicrobial secondary metabolites, and the induction of local or systemic defense responses (Poveda, 2021; Vos et al., 2014). Notably, *Trichoderma* can envelop and attach to pathogenic fungi upon contact, forming appressoria on their surfaces. This interaction facilitates the secretion of cell wall-degrading enzymes, directly targeting and killing the pathogens (Saldajeno et al., 2014). Compared with the application of AM fungi or *Trichoderma* alone, the synergistic effect of combining these two biocontrol agents enhanced the control of plant diseases. The combination of *Trichoderma* and AM fungi has proven effective in controlling diseases in *Solanum lycopersicum*. Mwangi et al. (2011) found that combined inoculation significantly increased the aboveground biomass of *S. lycopersicum* by 11.6% to 69.7% compared to the use of AM fungi or *Trichoderma* alone. Interactions between the AM fungus and *Trichoderma* enhanced plant growth and suppressed damping off of cucumber (*Cucumis sativus* L.) and *Fusarium* wilt in melon plants grown in seedling nurseries (Chandanie et al., 2009; Martínez-Medina et al., 2009). Tanwar et al. (2013) reported that the incidence of *S. lycopersicum* inoculated with *Fusarium oxysporum* was 70.0%. In contrast, the incidence in plants inoculated with AM fungi (*Funneliformis mosseae* and *Acaulospora laevis*) decreased to 20.0%. Further co-inoculation with one AM fungus and *Trichoderma viride* reduced the incidence to 10.0%, while complete inhibition of the disease was achieved with the combined application of two AM fungi and *Trichoderma*.

Currently, studies exploring biological control strategies for anthracnose in common vetch are limited; however, AM fungi and *Trichoderma* show significant potential for managing this disease. The present study aims to investigate the effectiveness and mechanisms of the AM fungus, *Glomus tortuosum*, and *Trichoderma longibrachiatum*, both individually and in combination, along with the chemical agent pyraclostrobin for controlling anthracnose in common vetch under greenhouse conditions. We hypothesize that the combined application of *G. tortuosum* and *T. longibrachiatum* will have a synergistic effect, leading to greater disease suppression than any of the treatments applied alone.

## 2 Materials and methods

### 2.1 Common vetch, AM fungus, *Trichoderma*, and pathogens

The common vetch utilized in this study was *V. sativa* var. Lanjian No. 2, cultivated by Lanzhou University. The AM fungus *G. tortuosum* (BGC-NM03A) was obtained from the Bank of Glomeromycota in China (BGC), maintained by the Beijing Academy of Agriculture and Forestry Sciences (BAAFS). The strain was isolated from the alfalfa-growing soil in Ejin Horo Banner, Inner Mongolia. *Glomus tortuosum* was propagated by culturing *Trifolium repens* in sterilized sand, with root sections cut to a length of 1 cm to serve as inoculum, containing approximately 50 spores per gram. *Trichoderma longibrachiatum* was isolated from alpine meadows on the Tibetan Plateau (Zhu et al., 2024). The pathogen *C. spinaciae* was isolated from anthracnose-infected tissue of common vetch collected from the experimental site in Shantang Village, Xiahe County, Gansu Province, China (Wang et al., 2019). The common vetch, AM fungus, *Trichoderma*, and pathogens were provided by the State Key Laboratory of Herbage Improvement and Grassland Agro-ecosystems, Lanzhou University, China.

### 2.2 Experimental design

**Figure 1:**
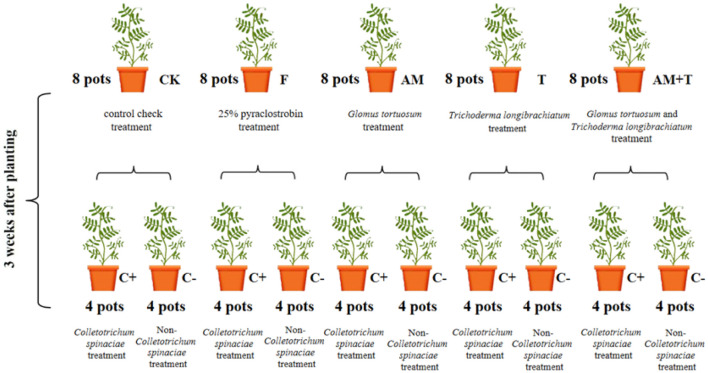
Design of the experiment and flowchart for each treatment.

The experiment utilized a factorial arrangement of 5 × 2 treatments. A solution of 25% pyraclostrobin (Henan Yintian Fine Chemical Co., Henan, China) (F) was sprayed at a concentration of 225 g·hm^-^², applying 20 mL per pot 3 days after the inoculation of pathogenic fungi. The treatments included *G. tortuosum* inoculation (AM), *T. longibrachiatum* inoculation (T), a combined inoculation of *G. tortuosum* and *T. longibrachiatum* (AM+T), and a control group with no inoculation (CK). This resulted in treatments divided into groups with pathogenic fungi inoculation and those without, yielding a total of 10 treatments. Each treatment was replicated four times, resulting in a total of 40 pots (**Figure 1**).

### 2.3 Experiment establishment

The soil was sieved through a 2-mm sieve and sterilized in an oven at 150°C for two cycles, each lasting 2 h, with a 24-h interval between cycles. The sand, also sieved through a 2-mm sieve, was heated in an oven at 170°C for 6 h. A mixture of the above sterilized soil and sand in a 1:3 ratio was prepared as the soil substrate, with each pot receiving 600 g of this substrate. For the treatments, 100 g of *G. tortuosum* and 100 g of *T. longibrachiatum* were measured and evenly distributed over the soil. The combined treatment (AM+T) received 50 g of each fungus, while the control (CK) and chemical (F) treatments were supplemented with equal amounts of sterilized mixed soil and sand. An additional 200 g of substrate was covered for each pot.

Seeds of common vetch cv. Lanjian No. 2 were sterilized by immersion in 75% alcohol for 3 min, followed by treatment with 1% sodium hypochlorite for 10 min. After rinsing the seeds five times with sterile water, the seeds were placed in sterile Petri dishes on filter paper and kept moist by daily watering with sterilized water. After 48 h of incubation, 12 seedlings were transplanted into pots, with 10 seeds per pot. A week later, eight plants displaying similar growth conditions were selected for further cultivation. Each pot received 150 mL of water every 3 days.

Three weeks after planting, the pathogen *C. spinaciae* was prepared for inoculation. The pathogen was scraped with a sterilized slide, filtered, and prepared into a spore suspension at a concentration of 1 × 10^6^ spores/mL. This suspension was inoculated onto the common vetch using the stabbing spray method, applying 200 mL per pot. The pots were then covered with black plastic bags for 48 h to promote infection. Two weeks after pathogen inoculation, disease incidence and disease index were recorded, and photosynthesis indicators were measured before harvest using a portable photosynthesis-fluorescence measurement system (GFS3000). The experiment was carried out in a greenhouse at Lanzhou University. Throughout the study, the photosynthetic photon flux density varied between 480 and 850 mmol/m²·s, while average temperatures ranged from 20°C to 28°C. The plants received tap water every 2 days until the soil reached a stable weight equivalent to 10% of its dry weight.

### 2.4 Plant harvest

Five weeks after emergence, the plants were harvested. The shoots were harvested and divided with three subsamples, and ~1 g from each pot was used for the measurement of the activities of plant defense enzyme activities: superoxide dismutase (SOD) activity was assessed using the nitrogen blue tetrazolium (NBT) photoreduction method, peroxidase (POD) activity was determined by the guaiacol method, catalase (CAT) activity was measured with the UV-absorbance method, and polyphenol oxidase (PPO) activity was evaluated using the catechol method (Gao et al., 2018). Additionally, ~1 g was used to determine the soluble sugar content with the anthrone method, proline content using the ninhydrin method, and malondialdehyde content through the trichloroacetic acid method. In addition, chlorophyll content was evaluated using the acetone extraction method (Li, 2000). The rest of the shoot was used for fresh weight, the fresh shoot was then dried in an oven for 48 h at 80°C, and the total dry weight was calculated with the ratio of fresh and dry weight.

The root was washed and divided into two subsamples, and ~0.1 g was used to determine the mycorrhizal colonization as described by Giovannetti and Mosse (1980). The rest of the fresh shoot was measured and then dried in an oven for 48 h at 80°C, and the total dry weight was calculated with the ratio of fresh and dry weight. The dried samples of the shoots and roots were crushed and used for the measurement of total nitrogen and phosphorus using a SmartChem 450 automatic chemical analyzer (AMS Alliance, Italy). Soil available phosphorus was measured by the molybdenum-antimony colorimetric method (Li, 2000). Soil neutral phosphatase, urease activity, and peroxidase activity were measured using the Hefei Lyle Biological Soil Enzyme Reagent Kit following the kit’s instructions.

### 2.5 Data analysis

All data were expressed as means with standard errors based on four replicates. Statistical comparisons between treatment and control groups were conducted using one-way analysis of variance (ANOVA) in SPSS 26.0 (SPSS Institute Inc., Chicago, IL, USA). Tukey’s HSD test was applied for all pairwise comparisons of treatment means at a significance level of *P <*0.05.

## 3 Results

### 3.1 Mycorrhizal colonization and *Trichoderma* in soils

**Figure 2:**
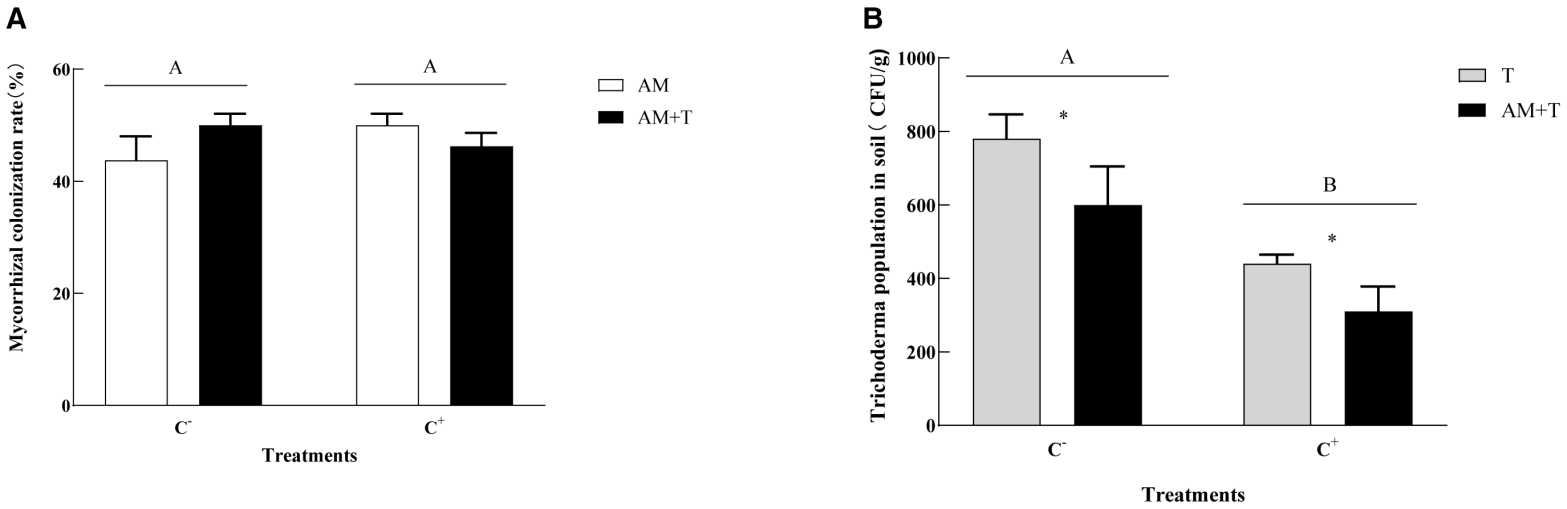
Mycorrhizal colonization rate **(A)** and *Colletotrichum spinaciae* population in the soil **(B)** under different treatments. Different uppercase letters above the bars indicate that there is no significant difference under different *Glomus tortuosum*-infected treatments **(A)** and there is a significant difference under different *Trichoderma longibrachiatum*-infected treatments **(B)**. C+, *C. spinaciae*-infected; C−, *C. spinaciae* un-infected; AM, *G. tortuosum* inoculation; T, *T. longibrachiatum* inoculation; AM+T, *G. tortuosum* and *T. longibrachiatum* inoculation. * indicate significant differences between treatments at P<0.05.

The inoculation of AM fungi formed typical arbuscular and hypha structures in the roots, with the infection rate ranging from 43.75% to 50.00%, with no significant differences among the treatments (**Figure 2**). Non-mycorrhizal structures were detected in the uninoculated (NM) root segments. The presence of disease reduced the spore numbers of *Trichoderma* in the soil. Compared to the non-pathogen-infected group, the population of *Trichoderma* decreased by 45.65% under pathogen infection (*P* < 0.05). The presence of AM fungi also reduced the *Trichoderma* spore numbers. In the co-inoculation treatment of AM fungi and *Trichoderma*, the *Trichoderma* spore numbers decreased by 25.62% compared to the treatment with *Trichoderma* alone (*P* < 0.05) (**Figure 2**).

### 3.2 Disease incidence and disease index

**Figure 3:**
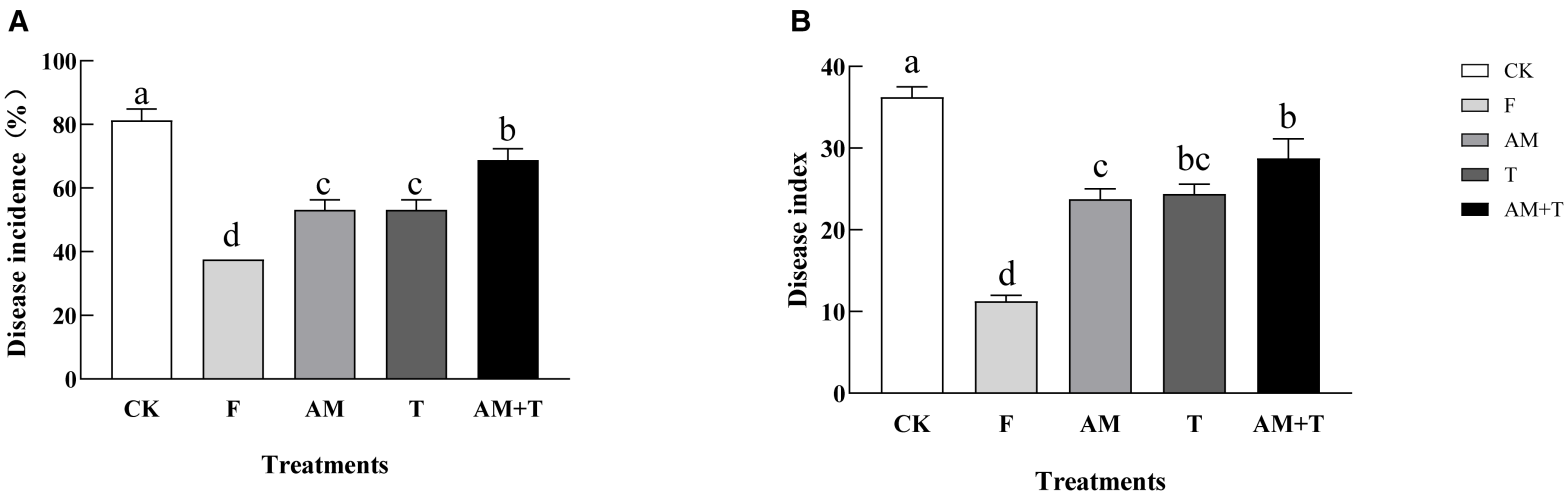
Disease incidence **(A)** and disease index **(B)** of common vetch infected by *Colletotrichum spinaciae* under different treatments. Different lowercase letters above the bars indicate that there is a significant difference at the *P* < 0.05 level among treatments. F, 25% pyraclostrobin sprayed; AM, *Glomus tortuosum* inoculation; T, *Trichoderma longibrachiatum* inoculation; AM+T, *G. tortuosum* and *T. longibrachiatum* inoculation.

The application of the fungicide 25% pyraclostrobin (F), the AM fungi *G. tortuosum* (AM), *T. longibrachiatum* (T), and the combination of AM fungi and *Trichoderma* significantly decreased the severity of common vetch anthracnose. Disease incidence was reduced by 53.85%, 34.62%, 34.62%, and 15.39%, respectively (*P* < 0.05), and disease index was reduced by 68.97%, 34.48%, 32.76%, and 20.69%, respectively (*P* < 0.05) (**Figure 3**).

### 3.3 Photosynthesis index

**Figure 4:**
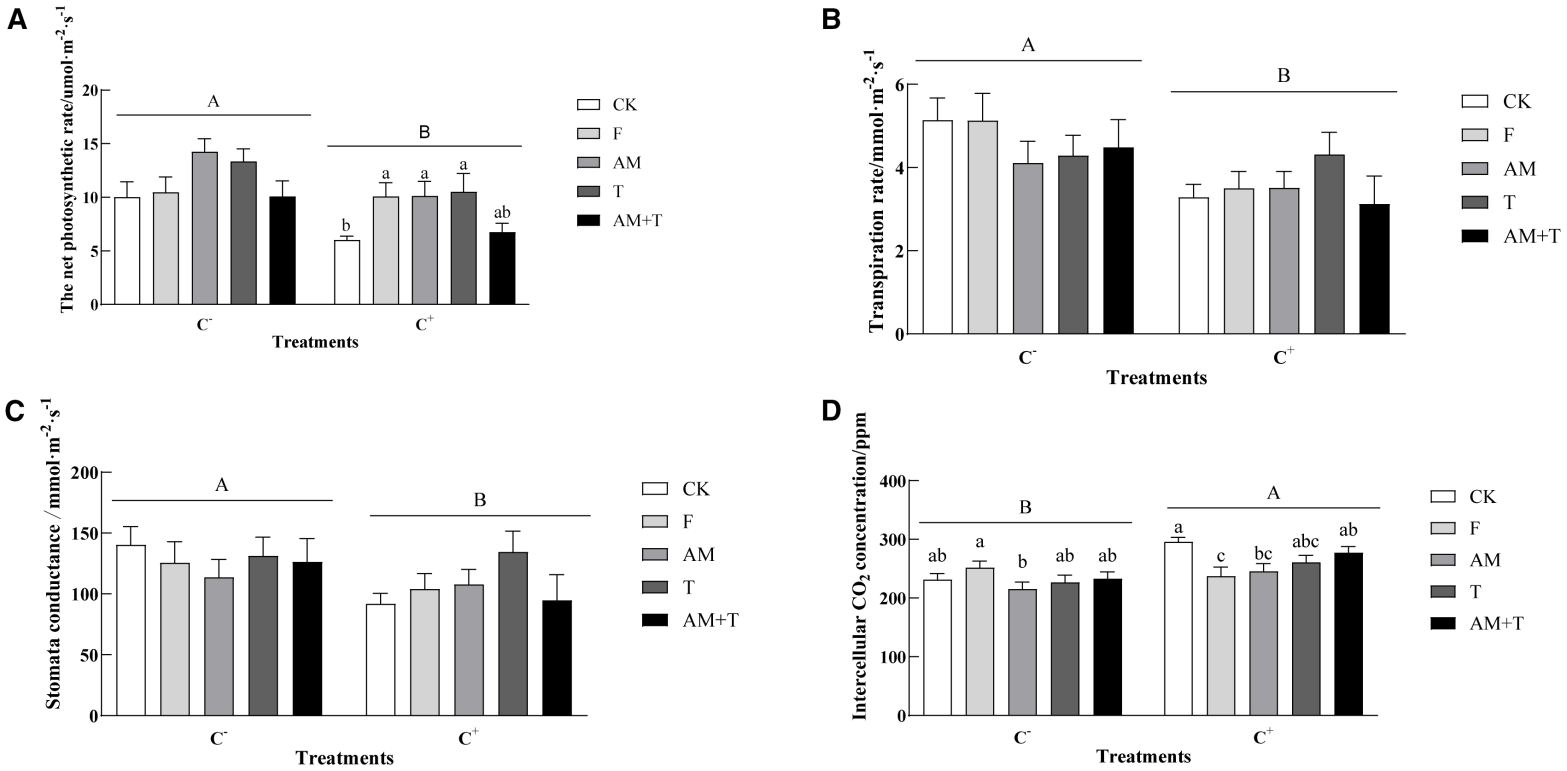
The net photosynthetic rate **(A)**, transpiration rate **(B)**, stomata conductance **(C)**, and intercellular CO_2_ concentration **(D)** of common vetch under different treatments. Different uppercase letters above the bars indicate that there is a significant difference between *Colletotrichum spinaciae*-infected (C+) and *C. spinaciae* un-infected (C−) treatments. Different lowercase letters above the bars indicate that there is no significant difference at the *P* < 0.05 level among treatments in C+ plant **(A)**, and there is a significant difference at the *P* < 0.05 level among treatments (**A, D**). F, 25% pyraclostrobin sprayed; AM, *Glomus tortuosum* inoculation; T, *Trichoderma longibrachiatum* inoculation; AM+T, *G. tortuosum* and *T. longibrachiatum* inoculation.

Anthracnose significantly affected the photosynthetic indices of common vetch. The net photosynthetic rate, transpiration rate, and stomatal conductance of common vetch were significantly reduced by the pathogen-infected treatment (C+), with decreases of 33.68%, 30.42%, and 19.50%, respectively, compared to plants under the non-pathogen-infected treatment (C−) (*P* < 0.05). Furthermore, the pathogen-infected treatment significantly increased the intercellular carbon dioxide concentration by 12.01% (*P* < 0.05). In the non-pathogen-infected treatment, there were no significant differences in net photosynthetic rates among the groups (*P* > 0.05). In the pathogen-infected treatment, the net photosynthetic rates under the fungicide, AM fungi, and *Trichoderma* treatments were significantly higher than the control, with increases of 67.16%, 67.94%, and 74.30%, respectively (*P* < 0.05). Regardless of whether the plants were infected or not, there were no significant differences in transpiration rates and stomatal conductance among the treatments (*P* > 0.05). In the non-pathogen-infected treatment, the intercellular carbon dioxide concentration of common vetch under the AM fungi treatment was 14.48% lower than that of the fungicide treatment group (*P* < 0.05). However, in the pathogen-infected treatment, the intercellular carbon dioxide concentrations of common vetch in the fungicide and AM fungi treatment groups were both lower than that of the control, by 19.83% and 17.07%, respectively (*P* < 0.05) (**Figure 4**).

### 3.4 The biomass of common vetch

**Figure 5:**
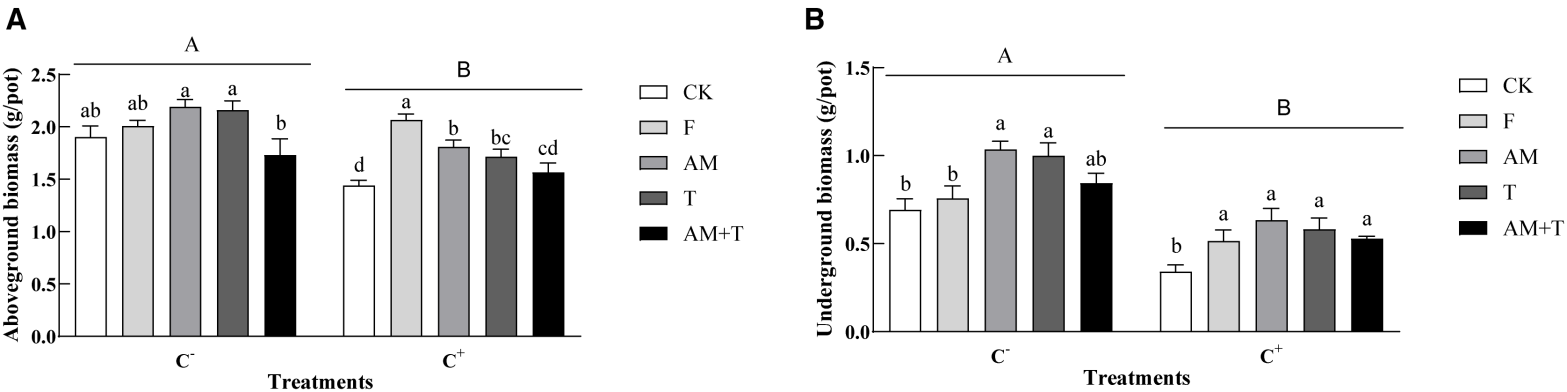
Aboveground biomass **(A)** and underground biomass **(B)** of common vetch under different treatments. Different uppercase letters above the bars indicate that there is a significant difference between *Colletotrichum spinaciae*-infected (C+) and *C. spinaciae* un-infected (C−) treatments. Different lowercase letters above the bars indicate that there is a significant difference at the *P* < 0.05 level among treatments. F, 25% pyraclostrobin sprayed; AM, *Glomus tortuosum* inoculation; T, *Trichoderma longibrachiatum* inoculation; AM+T, *G. tortuosum* and *T. longibrachiatum* inoculation.

Pathogen infection significantly decreased the growth of common vetch, with aboveground biomass decreasing by 13.96% and underground biomass by 39.98% (*P* < 0.05). AM fungi and *Trichoderma* did not affect the growth of common vetch under the non-pathogen-infected treatments (*P* > 0.05); however, their individual application resulted in significantly higher growth compared to the combined treatment of AM fungi and *Trichoderma*, with increases of 26.59% and 24.80%, respectively (*P* < 0.05). In addition, the application of AM fungi and *Trichoderma* significantly increased the underground biomass of common vetch by 49.71% and 44.47%, respectively (*P* < 0.05). Under the pathogen-infected treatment (C+), the application of fungicide, AM fungi, and *Trichoderma* significantly promoted the growth of plants, with aboveground biomass increases of 43.41%, 25.66%, and 19.09%, respectively (*P* < 0.05); the underground biomass also increased significantly by 51.76%, 86.02%, 70.99%, and 55.20%, respectively (*P* < 0.05) (**Figure 5**).

### 3.5 Nitrogen and phosphorus contents

Anthracnose significantly decreased nitrogen content in both the shoots and roots by 15.76% and 16.84%, respectively (*P* < 0.05). In the non-pathogen-infected treatment, there were no significant differences in nitrogen content in the aboveground and underground parts of common vetch (*P* > 0.05). In the pathogen-infected treatment, the nitrogen content in the shoots under the fungicide, AM fungi, and *Trichoderma* treatments was significantly higher than that of the control, with increases of 33.44%, 13.88%, and 12.12%, respectively (*P* < 0.05). The nitrogen content in the roots under the AM fungi treatment was 17.27% higher than that of the control (*P* < 0.05), while the nitrogen content in the roots under the other three treatments showed no significant difference compared to the control.

**Figure 6:**
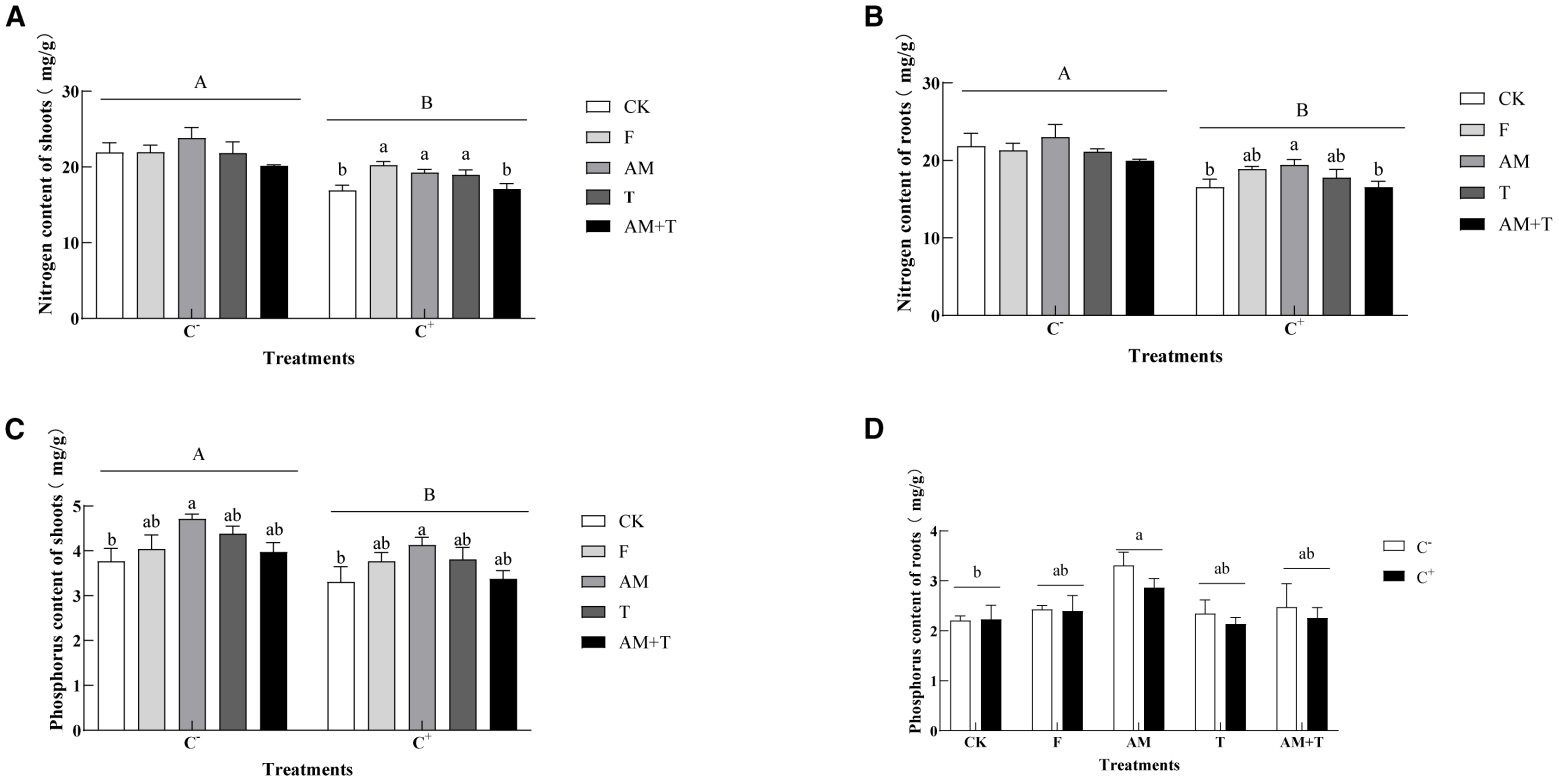
Nitrogen content of the shoots **(A)** and roots **(B)** and phosphorus content of the shoots **(C)** and roots **(D)** of common vetch under different treatments. Different uppercase letters above the bars indicate that there is a significant difference between *Colletotrichum spinaciae*-infected (C+) and *C. spinaciae* un-infected (C−) treatments (**A–C**). Different lowercase letters above the bars indicate there is a significant difference at the *P* < 0.05 level among treatments. F, 25% pyraclostrobin sprayed; AM, *Glomus tortuosum* inoculation; T, *Trichoderma longibrachiatum* inoculation; AM+T, *G. tortuosum* and *T. longibrachiatum* inoculation.

Anthracnose also significantly affected phosphorus content in the shoots of common vetch, resulting in an overall decrease of 11.96% after infection (*P* < 0.05), but it had no significant impact on phosphorus content in the roots (*P* > 0.05). Under the AM fungi treatment, phosphorus content in both the shoots and roots was significantly higher than that of the control, with increases of 25.01% and 39.21%, respectively (*P* < 0.05). In the other three treatments, with or without pathogen infection, there were no significant differences in phosphorus content in either the shoots or roots compared to the control (**Figure 6**).

### 3.6 Soil enzyme activities

**Figure 7:**
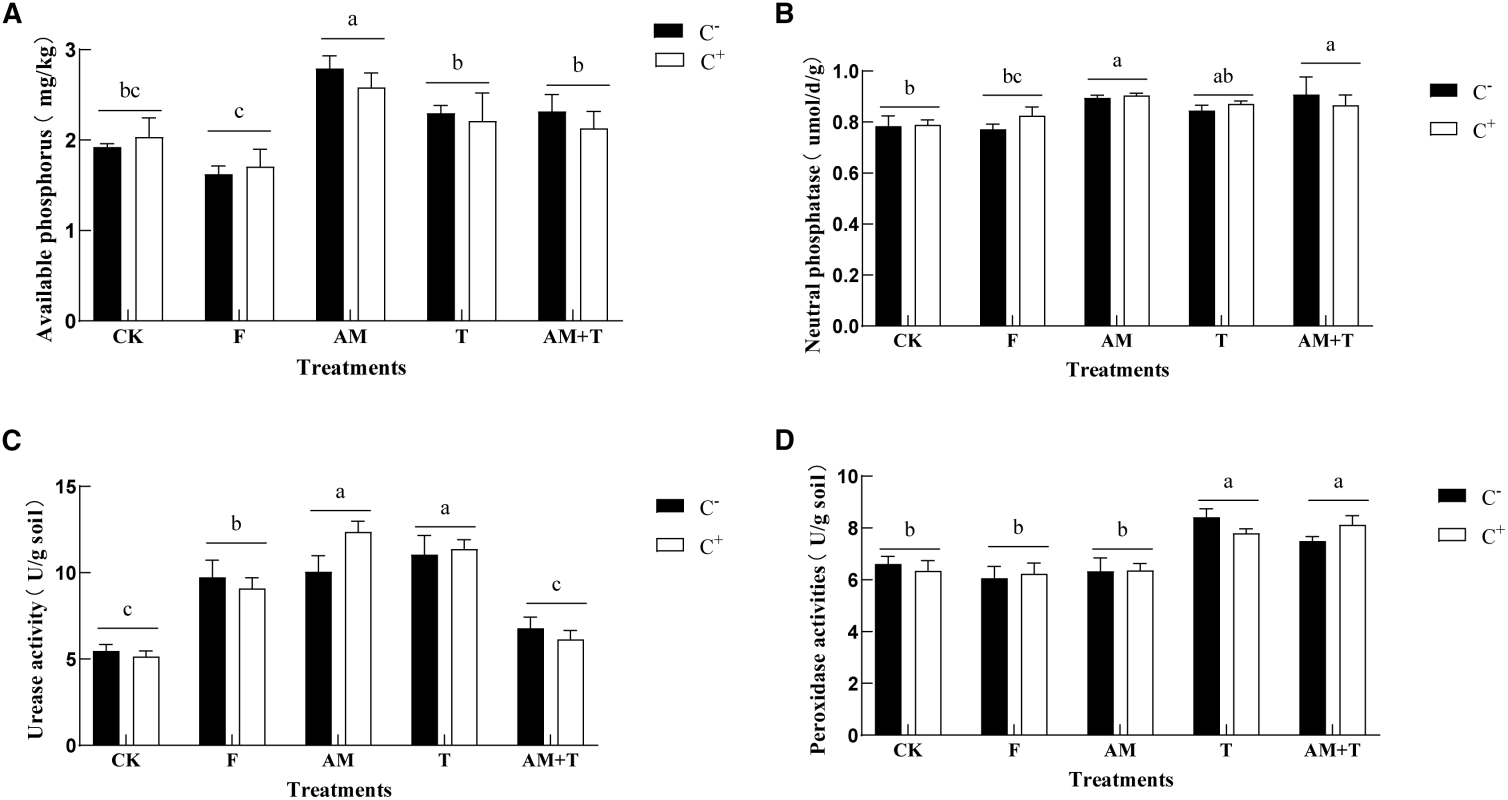
Soil available phosphorus **(A)** and neutral phosphatase **(B)**; urease activity **(C)** and peroxidase activity **(D)** under different treatments. Different lowercase letters above the group of bars means that there are significant differences among treatments. C+, *Colletotrichum spinaciae* infected; C−, *C. spinaciae* un-infected. F, 25% pyraclostrobin sprayed; AM, *Glomus tortuosum* inoculation; T, *Trichoderma longibrachiatum* inoculation; AM+T, *G. tortuosum* and *T. longibrachiatum* inoculation.

The infection with the anthracnose pathogen *C. spinaciae* did not affect soil enzyme activity. The urease activity in soil treated with the AM fungus and *Trichoderma* was significantly higher than that of the control group, showing an increase of 111.17% (*P* < 0.05) (**Figure 7**). Additionally, the peroxidase activity in the soil treated with *Trichoderma* alone or with the combination of AM and *Trichoderma* was 25.11% and 20.52% higher than the control, respectively (*P* < 0.05) (**Figure 7**). Furthermore, the neutral phosphatase activity in soils treated with AM fungi alone and the AM + *Trichoderma* combination was 14.45% and 12.77% higher than that of the control, respectively (*P* < 0.05) (**Figure 7**).

### 3.7 Chlorophyll, soluble sugars, proline, and malondialdehyde content

**Figure 8:**
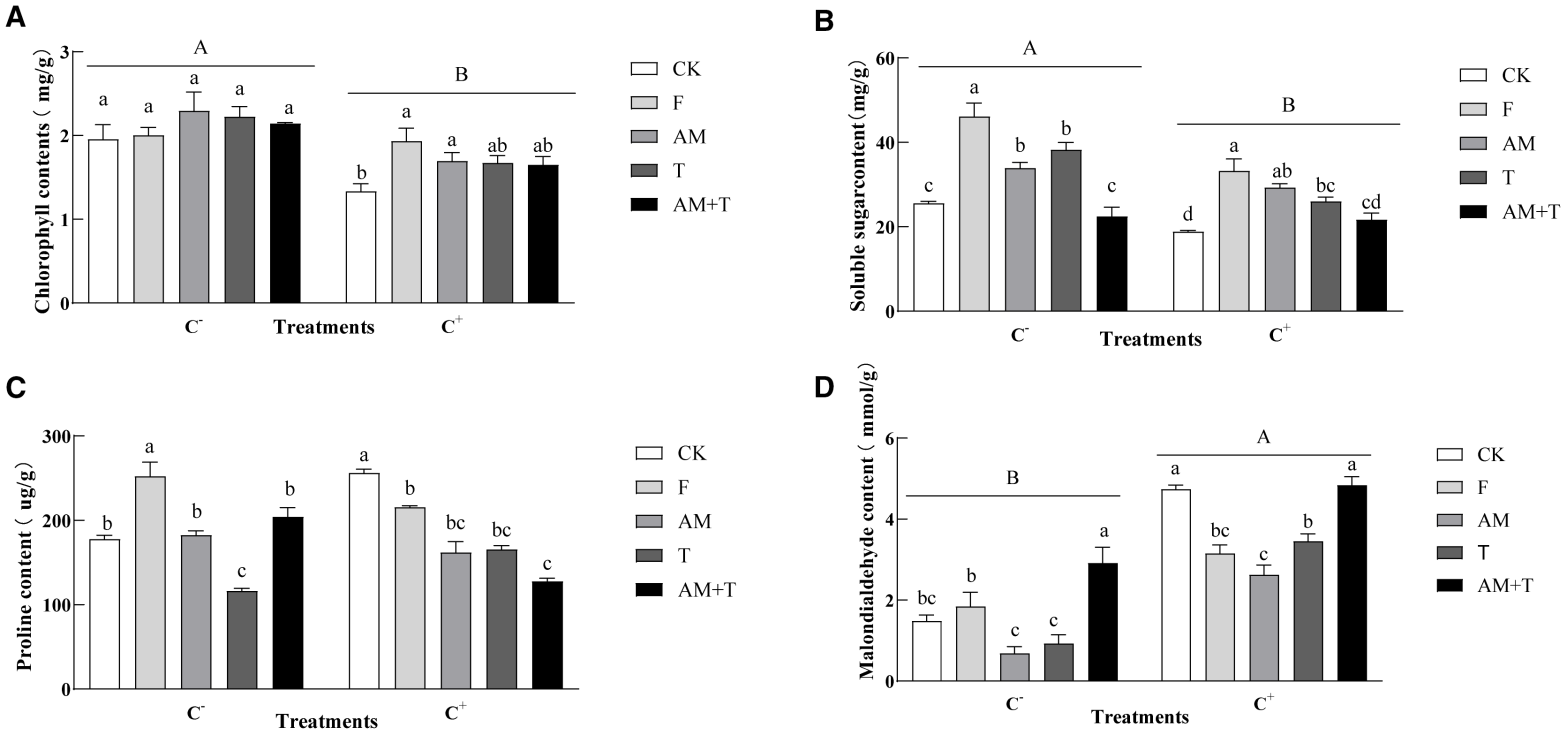
Chlorophyll **(A)**, soluble sugar content **(B)**, proline content **(C)**, and malondialdehyde content **(D)** of common vetch under different treatments. Different uppercase letters above the bars indicate that there is a significant difference between *Colletotrichum spinaciae*-infected (C+) and *C. spinaciae* un-infected (C–) treatments. **(A, B, D)** Different lowercase letters above the bars indicate that there is a significant difference at the *P* < 0.05 level among treatments. F, 25% pyraclostrobin sprayed; AM, *Glomus tortuosum* inoculation; T, *Trichoderma longibrachiatum* inoculation; AM+T, *G. tortuosum* and *T. longibrachiatum* inoculation.

Pathogen infection reduced chlorophyll by 21.99%. There were no significant differences among the treatments in the non-pathogen-infected plants. In contrast, under the pathogen-infected treatment, fungicides and AM fungi increased chlorophyll by 45.01% and 27.10% (*P* < 0.05). Pathogen infection also decreased the content of soluble sugars by 22.40% (*P* < 0.05), while it enhanced MDA content by 139.50% (*P* < 0.05). However, it did not have an impact on proline content. In the pathogen-infected treatment (C−), the application of fungicide, AM fungi, and *Trichoderma* increased soluble sugar content by 80.34%, 32.49%, and 49.37%, respectively (*P* < 0.05). In addition, the application of fungicides also increased the proline content by 61.05% (*P* < 0.05), while *Trichoderma* decreased the proline content by 31.46% (*P* < 0.05). The MDA content under the combined treatment of AM fungi and *Trichoderma* was 196.62% higher than CK (*P* < 0.05). In contrast, in pathogen-infected treatments (C+), the application of fungicide, AM fungi, and *Trichoderma* increased the soluble sugar content by 80.85%, 48.45%, and 38.28%, respectively (*P* < 0.05). The proline content in the control was significantly higher than in the other four treatments, with increases of 14.71%–58.31% (*P* < 0.05). The application of fungicide, AM fungi, and *Trichoderma* significantly reduced the malondialdehyde content by 44.03%, 43.15%, and 36.72%, respectively (*P* < 0.05) (**Figure 8**).

### 3.8 Plant defense enzyme activities

**Figure 9:**
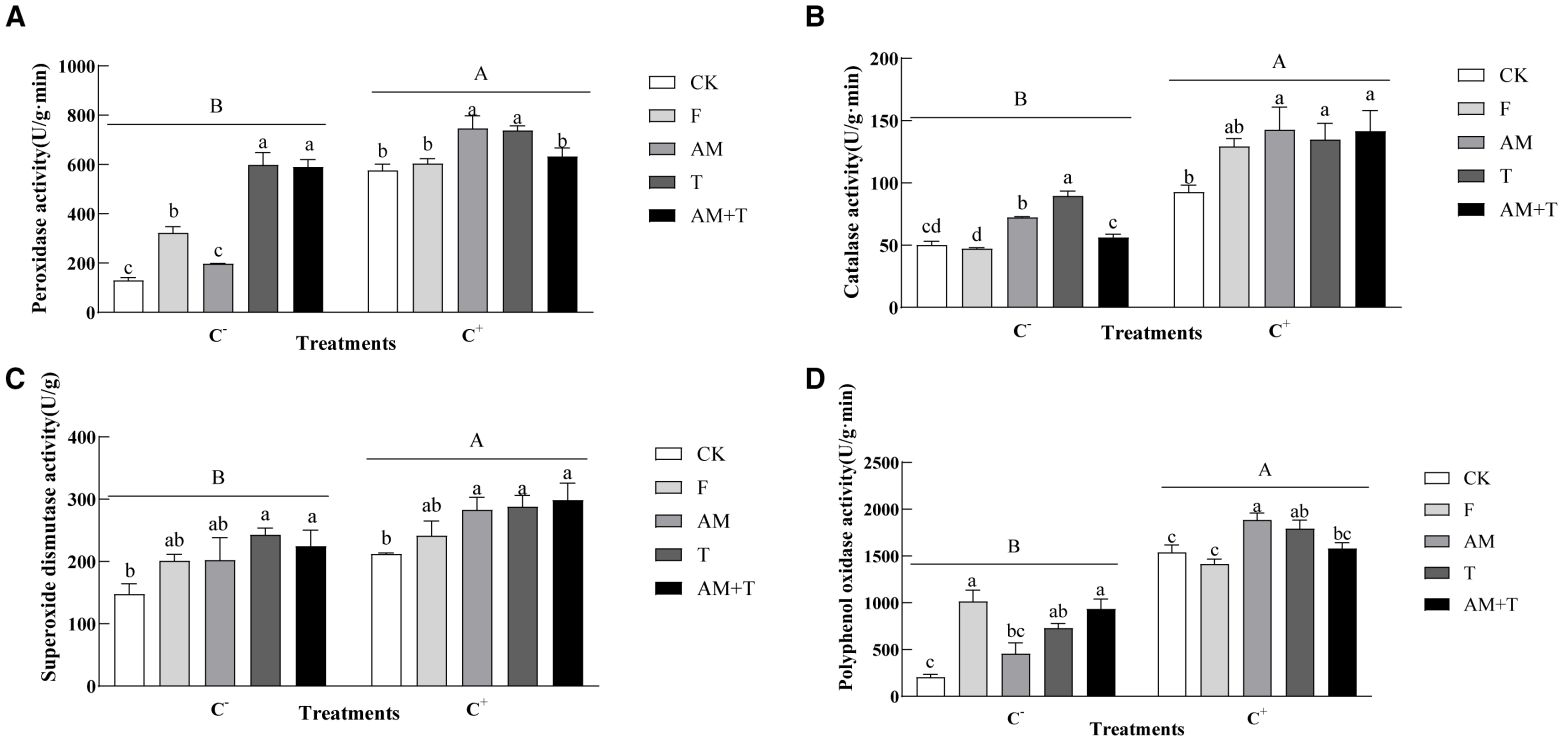
Peroxidase activity **(A)**, catalase activity **(B)**, superoxide dismutase activity **(C)**, and polyphenol oxidase activity **(D)** of common vetch under different treatments. Different uppercase letters above the bars indicate that there is a significant difference between *Colletotrichum spinaciae*-infected (C+) and *C. spinaciae* un-infected (C-) treatments. Different lowercase letters above the bars indicate that there is a significant difference at the *P* < 0.05 level among treatments. F, 25% pyraclostrobin sprayed; AM, *Glomus tortuosum* inoculation; T, *Trichoderma longibrachiatum* inoculation; AM+T, *G. tortuosum* and *T. longibrachiatum* inoculation.

Pathogen infection significantly increased defense enzyme activities, including POD, CAT, SOD, and PPO, with increasing rates of 79.54%, 103.28%, 29.90%, and 146.09% (*P* < 0.05), respectively. The application of fungicides, AM fungi, and *Trichoderma* significantly affected plant defense enzyme activities, whether in the presence or absence of pathogens. Under the non-pathogen-infected treatment, fungicides, AM fungi, and *Trichoderma* significantly increased the SOD activities by 148.50%, 361.41%, and 354.64% (*P* < 0.05), respectively; AM fungi and *Trichoderma* increased CAT activities by 44.295% and 78.80% (*P* < 0.05); the combination of AM fungi and *Trichoderma* significantly increased SOD activity by 64.28% and 51.90%, respectively (*P* < 0.05); and the AM fungi and *Trichoderma*, individually or in combination, increased PPO activities by 396.05%, 255.81%, and 357.08% (*P* < 0.05), respectively. Under the pathogen-infected treatment (C+), the application of AM fungi and *Trichoderma* significantly increased POD, CAT, SOD, and PPO activities by 23.57% and 22.10%, 27.12% and 26.76%, 21.54% and 19.33%, and 35.79% and 34.35%, respectively (*P* < 0.05) (**Figure 9**).

## 4 Discussion

The present study investigates the efficacy of the AM fungus, *G. tortuosum*, and *T. longibrachiatum*, both individually and in combination, in preventing and controlling anthracnose in common vetch, with 25% pyraclostrobin serving as a chemical control reference. AM fungi and *Trichoderma* species have demonstrated significant efficacy in managing plant anthracnose caused by *Colletotrichum* species. For instance, *G. intraradices* notably diminished anthracnose incidence in *Forsythia*, resulting in improved yields in mycorrhizal plants compared to non-mycorrhizal counterparts (Richter et al., 2011). Inoculation with *T. harzianum* significantly lessened the incidence of *C. gloeosporioides* in mango, decreasing its severity by 87.90% and demonstrating *Trichoderma*’s antagonistic potential against *C. graminicola* (Alvindia and Dionisio, 2018). *Trichoderma* exhibited an *in vitro* inhibition rate exceeding 70% against *C. graminicola*, and field applications showed marked reductions in seedling mortality and disease incidence across different growth stages, leading to increases in dry matter by 40.0% and grain yield by 23.8% compared to control plants (Vasanthakumari and Shivanna, 2014). In the current experiment, AM fungi and *Trichoderma* were effective in mitigating anthracnose in common vetch caused by *C. spinaciae*, significantly lowering both the incidence and severity of the disease.

Both the AM fungi and *Trichoderma* contribute positively to soil physicochemical properties, promoting root and aboveground growth to alleviate the negative impacts of pathogens (Zhu et al., 2024; Zheng et al., 2016). In this study, the application of AM fungi increased soil neutral phosphatase and urease activities, while *T. longibrachiatum* elevated soil catalase and urease activities. These increases were positively correlated with higher levels of soil available phosphorus and improved plant biomass. This finding aligns with previous research by Wang et al. (2007), who noted that AM fungi stimulate phosphatase and urease activities. The enhanced soil enzyme activity facilitated by AM fungi aids in the release of essential nutrients (Qin et al., 2020). Conversely, the application of *T. longibrachiatum* resulted in increased activities of soil urease, dehydrogenase, acid phosphatase, catalase, invertase, and acid protease (Mao and Jiang, 2021). This application also improved the plants’ ability to convert and utilize nitrogen and phosphorus from the soil.

Anthracnose negatively affects chloroplast content. Compared to uninoculated controls, pathogen-infected plants exhibited decreased net photosynthetic rates, lower stomatal conductance, reduced transpiration rates, and diminished chlorophyll a, b, and total chlorophyll content, while intercellular CO_2_ concentrations increased (Silva et al., 2020). Similar results were found in the current experiment, as the presence of the AM fungus and *Trichoderma* significantly enhanced chlorophyll content and photosynthetic capacity in diseased plants, aiding their resistance to pathogenic damage.

Plant responses to pathogenic attacks include the production of defense enzymes such as CAT, phenylalanine ammonia lyase (PAL), POD, PPO, and SOD (Li et al., 2018). Our results showed that both *G. tortuosum* and *T. longibrachiatum* were able to inhibit pathogen growth by inducing host resistance and producing defensive compounds. Inoculation with *G. fasciculatum* increased the levels of SOD, ascorbate peroxidase (APX), ascorbic acid (AA), and phenolics in plants, thereby enhancing resistance against anthracnose and promoting growth and biomass increase (Maya and Matsubara, 2013). *Trichoderma longibrachiatum* inhibited the mycelial growth of *C. graminicola* by up to 76.47%. *Trichoderma* increased the activities of SOD, POD, and PPO by 36.63%, 43.59%, and 40.96%, respectively, resulting in a 32.92% reduction in the anthracnose disease index while simultaneously exerting a growth-promoting effect (Manzar et al., 2021). In this experiment, both *G. tortuosum* and *T. longibrachiatum* stimulated plant resistance and significantly increased defense enzyme activity in common vetch, as evidenced by decreased malondialdehyde content and increased soluble sugar content, regardless of pathogen infection.

Although the combined application of *G. tortuosum* and *T. longibrachiatum* demonstrated effectiveness against anthracnose in common vetch, the overall impact was not fully satisfactory, potentially due to competitive interactions between the two agents. Previous research showed that AM fungi can influence the colonization of plant roots by plant growth-promoting rhizobacteria (PGPB), with AM fungi possibly inhibiting *T. longibrachiatum* activity and negatively affecting its population development, which may impair its growth-promoting and biocontrol effects (Manzar et al., 2021).

In addition to the AM fungus and *Trichoderma*, various biocontrol agents exhibiting strong antagonistic effects against anthracnose have been documented. For instance, sterile culture filtrate, crude proteins, crude lipopeptides, and volatile compounds from *Bacillus subtilis* exhibited strong antagonism against *C. gloeosporioides* (Huang et al., 2020). Furthermore, *Stenotrophomonas rhizophila* inhibited mycelial growth and spore germination of the pathogen *C. gloeosporioides* through the production of volatile compounds, nutrient competition, and lytic enzymes, successfully reducing anthracnose incidence in tomato by 95% (Reyes-Perez et al., 2019). Additionally, *Bacillus amyloliquefaciens* demonstrated strong antagonism against *C. truncatum*, the causal agent of alfalfa anthracnose, achieving 60% inhibition of mycelial growth and complete inhibition of conidial germination and displaying 82.59% efficacy against alfalfa anthracnose under greenhouse conditions (Hu et al., 2020). Timely evaluation and field testing of these biocontrol agents are critical for their potential commercialization in agricultural applications.

Our study also examined the effectiveness of pyraclostrobin, which exhibited significant inhibitory effects on anthracnose caused by *Colletotrichum* spp., with field trials revealing efficacy rates ranging from 41.14% to 48.91% against *C. gloeosporioides*. Furthermore, its effectiveness against *C. fructicola* reached 66.70% (Nam et al., 2014). The application of 25% pyraclostrobin (150 g·hm^-^²) demonstrated superior efficacy against anthracnose in alfalfa, achieving a reduction in disease index and a corresponding yield increase of 51.14%. Pyraclostrobin differentially diminished anthracnose severity caused by *Colletotrichum acutatum* and *C. lindemuthianum* (Gao et al., 2017; Rodriguez-Salamanca et al., 2015; Gillard and Ranatunga, 2013). In this study, 25% pyraclostrobin was also effective against anthracnose in common vetch, significantly reducing the disease index while increasing forage yield.

## 5 Conclusion

The present study shows that under greenhouse conditions, both individual and combined applications of the AM fungus *G. tortuosum* and *T. longibrachiatum* demonstrate significant preventive effects against anthracnose in common vetch. These biocontrol agents not only protect against the invasion of anthracnose by inducing systemic resistance and activating defense enzyme activities but also stimulate soil enzyme activity, enhancing nutrient absorption in common vetch. As a result, the co-application of the AM fungus and *Trichoderma* increases the resistance of common vetch to anthracnose disease. This study highlights the importance of implementing biological control methods to improve overall plant health and resilience against pathogens. Specifically, the use of the AM fungus and *Trichoderma* enhances disease resistance in common vetch.

## Data Availability

The original contributions presented in the study are included in the article/supplementary material. Further inquiries can be directed to the corresponding authors.
